# g-C_3_N_4_ Modified by *meso*-Tetrahydroxyphenylchlorin for Photocatalytic Hydrogen Evolution Under Visible/Near-Infrared Light

**DOI:** 10.3389/fchem.2020.605343

**Published:** 2020-11-06

**Authors:** Yanfei Liu, Zhen Ma

**Affiliations:** ^1^Shanghai Key Laboratory of Atmospheric Particle Pollution and Prevention (LAP^3^), Department of Environmental Science and Engineering, Fudan University, Shanghai, China; ^2^Shanghai Institute of Pollution Control and Ecological Security, Shanghai, China

**Keywords:** g-C_3_N_4_, *meso*-tetrahydroxyphenylchlorin, photocatalytic, hydrogen evolution, visible/near-infrared light

## Abstract

A new photocatalyst denoted as mTHPC/pCN was prepared by modifying protonated graphitic carbon nitride (pCN) by *meso*-tetrahydroxyphenylchlorin (mTHPC). Relevant samples were characterized via various methods including zeta potential measurements, X-ray diffraction, Fourier transform infrared spectroscopy, X-ray photoelectron spectroscopy, N_2_ adsorption–desorption, transmission electron microscopy, ultraviolet-visible–near-infrared spectroscopy, electrochemical impedance spectroscopy, photocurrent response measurements, electron spin resonance spectroscopy, and phosphorescence spectroscopy. Compared with pCN, mTHPC/pCN shows enhanced absorption in the visible and near-infrared regions and thus higher photocatalytic activity in hydrogen evolution. A possible mechanism for mTHPC/pCN is proposed.

## Introduction

Graphitic carbon nitride (g-C_3_N_4_) is a new type of photocatalyst with unique physicochemical characteristics (Zheng et al., 2015). The π-conjugated system of g-C_3_N_4_ allows for the transfer of charge carriers, and a band gap of around 2.7 eV allows it to work under visible (VIS) light (Wang et al., 2009; Ong et al., 2017). In addition, g-C_3_N_4_ is thermally and chemically stable. It can be prepared by the thermal polycondensation of inexpensive nitrogen-containing carbon-based precursors such as thiourea, melamine, urea, cyanamide, and dicyandiamide without difficulty (Panneri et al., 2017).

However, the low efficiency in VIS light absorption, high recombination rate of photogenerated electrons and holes, low conductivity, and low specific surface areas (SSAs) of g-C_3_N_4_ may limit its photocatalytic performance (Zou et al., 2016; Mishra et al., 2019). g-C_3_N_4_ nanorods/nanotubes (Li et al., 2015; Liu et al., 2017), nanasheets (Zhang J. S. et al., 2015; Murugesan et al., 2019), and porous structures (Zeng et al., 2016; Liu M. J. et al., 2019) have been developed. Metal elements (e.g., Ag; Ge et al., 2011, Cu; Fan et al., 2016, Au; Caux et al., 2019, Pt; Zhou et al., 2019) and non-metal elements (e.g., C; Zhao et al., 2015, N; Fang et al., 2015, P; Ran et al., 2015, Br; Lan et al., 2016, O; Wei et al., 2018, S; Xiao et al., 2020) have been doped into g-C_3_N_4_. In addition, g-C_3_N_4_-based heterojunctions (e.g., Bi_2_O_2_CO_3_/g-C_3_N_4_; Wang Z. Y. et al., 2016, CoTiO_3_/g-C_3_N_4_; Ye et al., 2016, Ag_2_MoO_4_/g-C_3_N_4_; Zhang and Ma, 2017b, C/g-C_3_N_4_; Shen et al., 2017, Ag_6_Mo_10_O_33_/g-C_3_N_4_; Zhang and Ma, 2017a, MoS_2_/g-C_3_N_4_; Liu Y. Z. et al., 2018, Bi_3_O_4_Cl/g-C_3_N_4_; Che et al., 2018, TiO_2_/g-C_3_N_4_; Tao et al., 2019, WO_3_/g-C_3_N_4_; Fu et al., 2019, CdS/g-C_3_N_4_; Qiu et al., 2020, ZnO/g-C_3_N_4_; Gao et al., 2020, Ba_5_Nb_4_O_15_/g-C_3_N_4_; Wang et al., 2020, Co_3_(PO_4_)_2_/g-C_3_N_4_; Shi et al., 2020, Cs_3_Bi_2_I_9_/g-C_3_N_4_; Bresolin et al., 2020) have been developed to enhance the photocatalytic performance. However, few studies have aimed at extending the light absorption range of g-C_3_N_4_ to the near-infrared (NIR) region.

In the total solar spectrum, the ultraviolet (UV) light (λ < 400 nm), VIS light (400 < λ < 700 nm), and NIR light (λ > 700 nm) account for ~5, 43, and 52%, respectively (Li et al., 2016). Therefore, the development of g-C_3_N_4_-based photocatalysts that can absorb NIR light is important. Photosensitizers are the general term of molecules that can absorb light and transfer energy to other materials. Some researchers modified g-C_3_N_4_ with photosensitizers such as phthalocyanine (Zhang et al., 2014), a combination of organic dye and zinc phthalocyanine derivative (Zhang X. H. et al., 2015), μ-oxo dimeric iron (III) porphyrin (Wang D. H. et al., 2016), zinc phthalocyanine (Liu Q. W. et al., 2018), mesotetrakis (carboxyphenyl) porphyrins (Da Silva et al., 2018), copper octacarboxyphthalocyanine (Ouedraogo et al., 2018), zinc phthalocyanine derivative (Zeng et al., 2019), multiporphyrin (Yang et al., 2019), zinc (II) *1, 8(11), 15(18), 22(25)*-tetrakis (4-carboxylphenoxy) phthalocyanine (α-ZnTcPc) (He et al., 2019), porphyrin (Tian et al., 2019), Chlorin e6 (Ce6) (Liu et al., 2020a), 3,4,9,10-perylenetetracarboxylic acid anhydride (PTCDA) (Yuan et al., 2020), tetra (4-carboxyphenyl) porphyrin iron (III) chloride (FeTCPP) (Zhang et al., 2020), protoporphyrin (Pp) (Liu et al., 2020b), and naphthalimide-porphyrin (Li L. L. et al., 2020). However, more examples in this regard are needed because this is a very interesting topic.

*meso*-Tetrahydroxyphenylchlorin (mTHPC or temoporfin) is an NIR light–absorbing photosensitizer used for clinical applications and photodynamic therapy (Navarro et al., 2014). In addition, mTHPC is a second-generation photosensitizer that shows some favorable characteristics under NIR light irradiation (Hinger et al., 2016). For example, multiwalled carbon nanotubes were modified with mTHPC for cancer treatment (Marangon et al., 2016). Polymeric micelles were modified with mTHPC for treating cardiovascular diseases (Wennink et al., 2017). Gold nanoparticles were modified with mTHPC for cancer therapeutic (Haimov et al., 2018). Poly(d,l-lactide-co-glycolide) acid nanoparticles were modified with mTHPC for *in vitro* photodynamic therapy (Boeuf-Muraille et al., 2019). However, to the best of our knowledge, g-C_3_N_4_ was not modified by mTHPC for photocatalysis.

**Figure 1:**
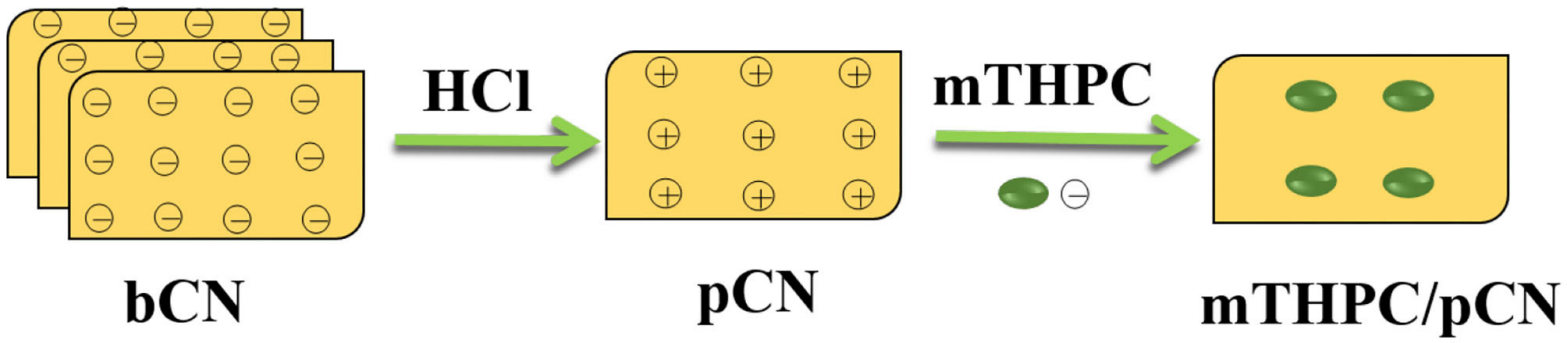
Scheme showing the synthesis of mTHPC/pCN.

Supplementary Figure 1 shows the chemical structures of mTHPC and bulk graphitic carbon nitride (bCN). mTHPC has many hydroxyl (–OH) groups, and bCN has many –C–N– groups, making the surfaces of both materials negatively charged (Supplementary Figure 2) and thus difficult to combine with each other. Herein, protonated graphitic carbon nitride (pCN) was obtained by treating bCN with hydrochloric acid (HCl) solution (Xie et al., 2018). The surface zeta potential of pCN is positively charged (Supplementary Figure 2), so the negatively charged mTHPC may be combined with pCN to yield a composite photocatalyst (Figure 1) that can work more efficiently under VIS light and NIR light.

## Experimental Section

### Synthesis of Bulk Graphitic Carbon Nitride (bCN)

bCN was prepared by high-temperature calcination in a muffle furnace. The details are as follows: 12 g melamine was placed in a 50 mL quartz crucible with a lid, and then the quartz crucible was placed in a muffle furnace. The heating rate was set to be 5°C · min^−1^. The muffle furnace was heated to 550°C, and the temperature was held for 2 h (Cui et al., 2018a). After the muffle furnace was cooled down, the remaining powders (bCN) were collected and ground for further use.

### Preparation of pCN

pCN was obtained by treating bCN with HCl solution. The details are as follows: 1 g bCN was placed in 200 mL HCl solution (1 mol · L^−1^), and the slurry was magnetically stirred at room temperature for 4 h (Cui et al., 2018b). Subsequently, the sediment was collected by high-speed centrifugation, washed with deionized water three times, and dried at 80°C for 12 h. The obtained powders (pCN) were ground for further use.

### Preparation of mTHPC/pCN and mTHPC/bCN

mTHPC/pCN was synthesized as follows: 0.5 g pCN was put into 200 mL deionized water, and then 0.05 g mTHPC was added, and the slurry was subjected to magnetic stirring for 2 h at room temperature. Subsequently, the sediment was collected by high-speed centrifugation, washed three times by deionized water, and dried at 80°C for 12 h. The obtained powders (mTHPC/pCN) were collected. In addition, a reference sample denoted as mTHPC/bCN was prepared under the same experimental conditions.

### Characterization

The surface zeta potential data were obtained from a Zetasizer Nano ZS device (Malvern Instruments). X-ray diffraction (XRD) data were recorded using a Bruker Advanced D8 (Bruker Corp., Germany) instrument. Fourier transform infrared (FTIR) spectra were recorded by a Nicolet Nexus 470 instrument (Nicolet Instrument Corp., USA). X-ray photoelectron spectra (XPS) were analyzed by an ESCALAB 250 XPS instrument. Nitrogen (N_2_) adsorption–desorption were determined by a Tristar 3000 analyzer. Transmission electron microscopy (TEM) images were taken using a JEM-2100F microscope (JEOL, Japan). UV-VIS-NIR absorption spectra were measured by a Cary 5000 spectrophotometer. Electrochemical impedance spectra (EIS) and photocurrent response curves were obtained through a CHI660C electrochemical workstation. Electron spin resonance (ESR) spectra were recorded by a Bruker model A300 spectrometer at room temperature. Phosphorescence spectra were tested on a Hitachi F-4600 spectrometer under an excitation wavelength of 808 nm.

### Photocatalytic Hydrogen Evolution

Photocatalytic hydrogen generation experiments were carried out as follows: 10 mg photocatalyst was placed in a 150 mL quartz reactor, 18 mL H_2_PtCl_6_ solution (0.045 mg · mL^−1^) was added, and then 2 mL triethanolamine (TEOA) was added. Subsequently, the mixed system was sonicated in an ultrasonic machine for 10 min to allow for the even dispersion of the photocatalyst and then purged with N_2_ for 30 min to remove air from the solution and the reactor. During photocatalytic process, the temperature of the photocatalytic reaction was controlled at about 12°C by using circulating cooling water, and a 300-W xenon (Xe) lamp was used as light source. A filter was used to get VIS-NIR light (λ > 420 nm). Another filter was used to get NIR light (λ > 780 nm).

The distance from the light source to the reaction system was 4 cm. The optical power density of the light source was 120 mW · cm^−2^ under VIS-NIR light irradiation and 10 mW · cm^−2^ under NIR light irradiation. The mixed gas composed of H_2_ produced by photocatalysis and N_2_ in the quartz reactor was automatically collected and analyzed using a gas chromatograph (GC7600, Tian Mei) every 1 h.

## Results and Discussion

### XRD and FTIR Spectra

**Figure 2:**
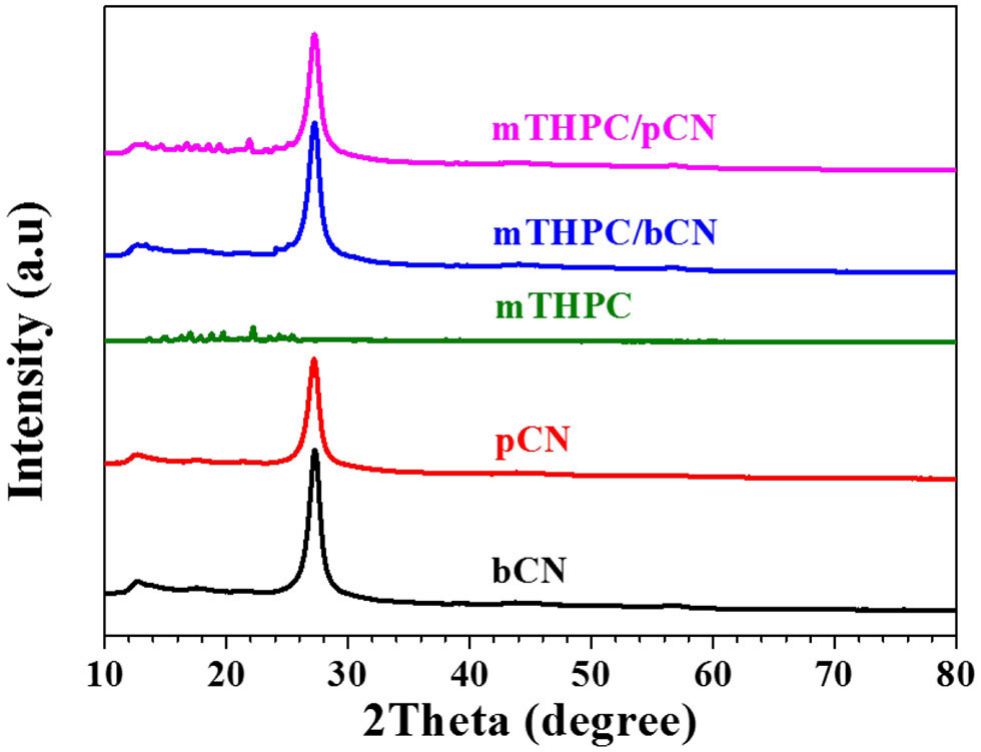
XRD patterns of samples.

Figure 2 shows the XRD data. The peak of bCN at 13.4° is attributed to the (100) plane (Inagaki et al., 2019; Qi et al., 2019), and the peak at 27.3° is attributed to the (002) plane (Liu L. et al., 2018; Lee et al., 2019). pCN has the same characteristic peaks as bCN, but the intensity of these peaks decreases to some extent, probably due to the delamination of bCN following the treatment of bCN by HCl solution (Liang et al., 2015; Prabavathi and Muthuraj, 2019). mTHPC shows some weak peaks in the range of 12 to 26° (Yuan et al., 2015a). The peaks of mTHPC are not observed in mTHPC/bCN. However, the peaks of mTHPC can be observed in mTHPC/pCN, indicating the uptake of mTHPC on pCN. In any case, bCN, pCN, mTHPC/bCN, and mTHPC/pCN all show typical g-C_3_N_4_ patterns.

**Figure 3:**
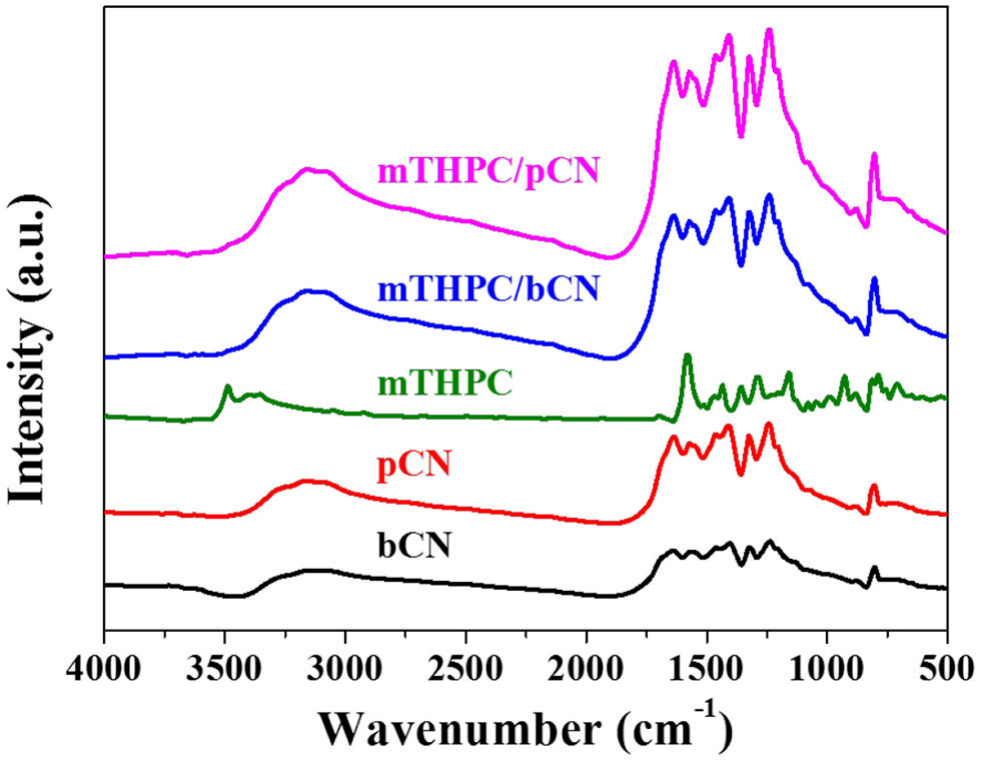
FTIR spectra of samples.

Figure 3 shows the FTIR spectra of samples. The characteristic peaks of bCN at 800 cm^−1^ is ascribed to the tri-s-triazine ring units (Zhang et al., 2010; Wang et al., 2019); the broadband peaks at 1,200 to 1,700 nm^−1^ are attributed to the C–N heterocycles (Liu Q. et al., 2016), and the peaks at 3,000 to 3,500 nm^−1^ are attributed to the hydroxyl groups (O–H) and free amino groups (N–H) (Hang et al., 2017; He et al., 2020). pCN has the same characteristic peaks as bCN, but the intensities of these peaks increase to some extent. mTHPC shows peaks at 700 to 1,700 nm^−1^, corresponding to (N–H), (C–H), (C=C), and (C=N) (Da Silva et al., 2018). The peaks at 3,250 to 3,600 nm^−1^ of mTHPC are attributed to the N–H bonds (Yuan et al., 2015b). Although the peaks of mTHPC are not clearly observed in mTHPC/bCN and mTHPC/pCN, all the characteristic peaks of g-C_3_N_4_ in mTHPC/pCN are the strongest. This may be because mTHPC is loaded on the surface of pCN, which enhances the infrared absorption. A similar trend (enhanced IR absorption) is also seen in the Ce6/pCN (Liu et al., 2020a) and Pp/pGCN (Liu et al., 2020b) systems.

### XPS Spectra

**Figure 4:**
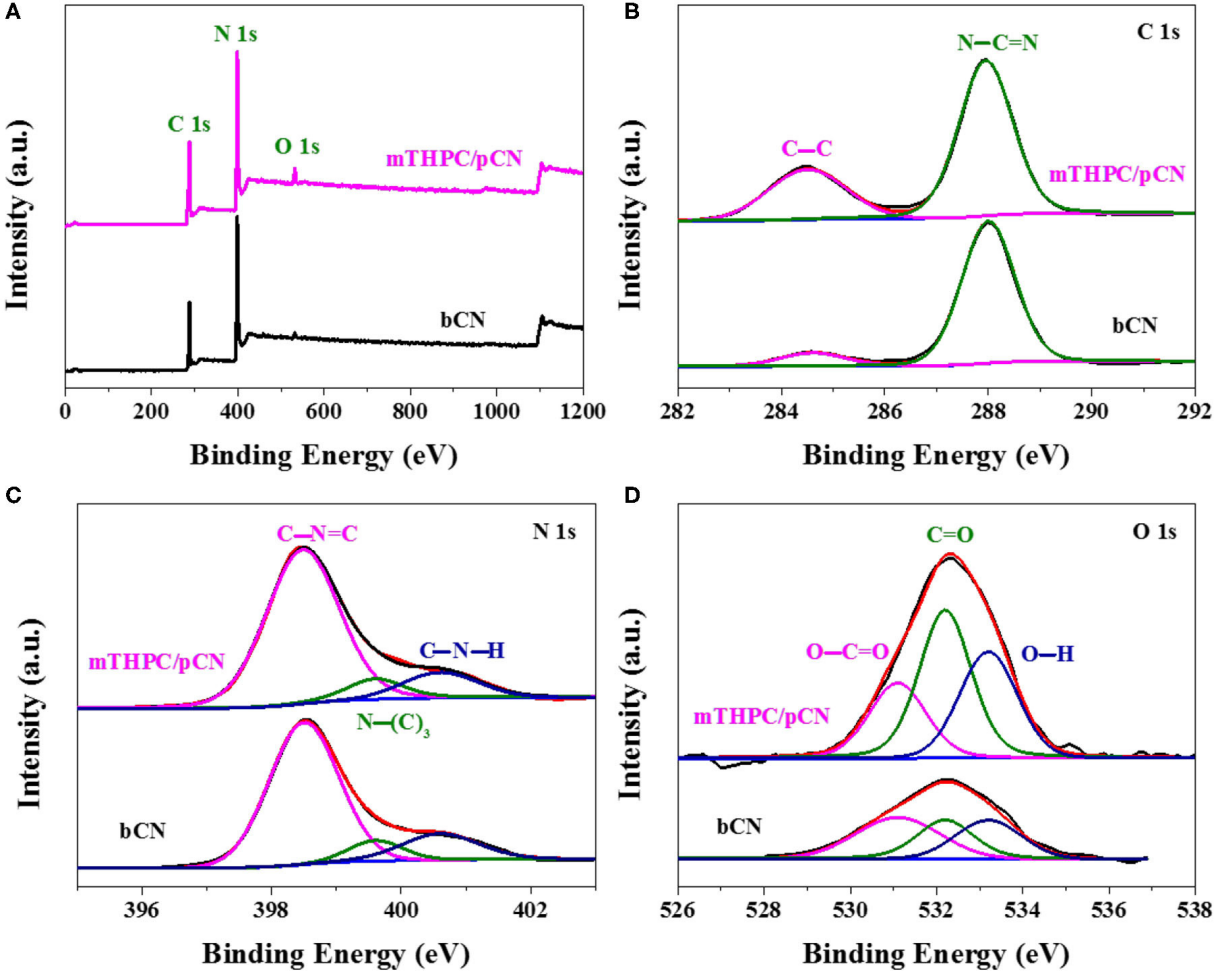
The survey XPS spectra **(A)**, the high-resolution XPS spectra of C 1s **(B)**, N 1s **(C)**, and O 1s **(D)** for bCN and mTHPC/pCN.

Figure 4 shows the XPS spectra of samples. In the survey XPS spectra of bCN and mTHPC/pCN (Figure 4A), the peaks of C 1s, N 1s, and O 1s can be obviously observed (Naseri et al., 2017; Zada et al., 2019). Figure 4B shows the high-resolution XPS spectra of C 1s. The two peaks at 284.5 and 287.9 eV are assigned to (C–C) and (N–C=N), respectively (Jiang et al., 2017; Liu X. C. et al., 2020). Figure 4C shows the high-resolution N 1s XPS spectra. The three peaks at 398.5, 399.6, and 400.6 eV are assigned to (C–N=C), (N–(C)_3_), and (C–N–H), respectively (Sun and Liang, 2017; Sun et al., 2020). Figure 4D shows the high-resolution O 1s XPS spectra. The three peaks at 531.1, 532.2, and 533.2 eV are due to O–C=O, C=O, and O–H, respectively (Teng et al., 2017; Zhang et al., 2017). Compared with bCN, the O 1s peaks (especially at 532.2 eV) of mTHPC/pCN are significantly enhanced, probably due to the oxygen-containing group in mTHPC.

### N_2_ Adsorption–Desorption and TEM Analysis

Supplementary Figure 3 shows the N_2_ adsorption–desorption data. The N_2_ adsorption and desorption isotherms of samples (Supplementary Figure 3A) can be classified as type IV isotherms, signifying the presence of mesopores (2–50 nm) (Qin and Zeng, 2017). The hysteresis loops of samples belong to H3 type, indicating the existence of slit-type mesopores formed by the irregular accumulation of g-C_3_N_4_ nanosheets (Ding et al., 2017). Compared with bCN, the adsorption volume of pCN appears significantly enhanced and this trend may be due to the nanosheet structure caused by the delamination treatment of bCN in HCl solution. mTHPC/pCN well maintains the enhanced adsorption volume.

Supplementary Figure 3B shows that the four samples have the wide pore size distribution (2–70 nm), further indicating the presence of mesopores (Qiu et al., 2017). It is worth noting that the pore size distribution curve of pCN has a clear peak at 2 to 5 nm, indicating that the porous structure may be caused by HCl solution, and mTHPC/pCN well maintains the porous structure.

Supplementary Table 1 shows the SSA and pore volume of samples. The SSA and pore volume of bCN are 12.5 m^2^ · g^−1^ and 0.063 cm^3^ · g^−1^, respectively. pCN has the largest SSA and pore volume of 40.3 m^2^ · g^−1^ and 0.163 cm^3^ · g^−1^, probably due to the delamination treatment of bCN by HCl solution. mTHPC/pCN well maintains a larger SSA and pore volume of 30.8 m^2^ · g^−1^ and 0.126 cm^3^ · g^−1^, respectively.

Supplementary Figure 4 shows the TEM images of samples. bCN has the irregularly thick bulk structure (Yu et al., 2017). However, pCN shows the typical two-dimensional nanoflakes and porous structure, consistent with the pore size distribution curve (Mamba and Mishra, 2016). mTHPC/pCN well maintains the ultrathin holey nanosheet structure of pCN.

### UV-VIS-NIR Absorption Spectra

**Figure 5:**
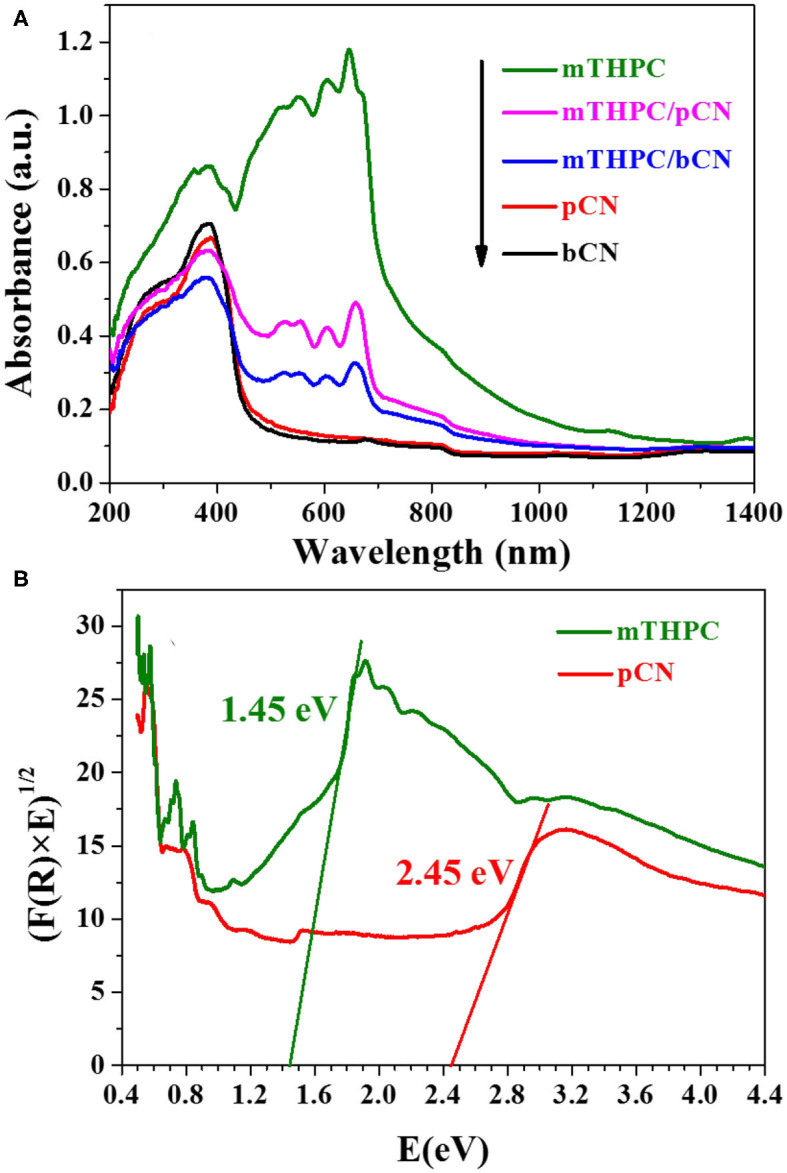
The UV-VIS-NIR absorption spectra of photocatalysts **(A)** and the calculated energy band gaps (*E*_g_) of mTHPC and pCN **(B)**.

Figure 5A shows the UV-VIS-NIR absorption spectra of photocatalysts. bCN and pCN only show obvious absorption capacity in the UV light and VIS light regions (Liu S. Z. et al., 2016) and have almost no absorption capacity in the NIR light region (λ > 780 nm). mTHPC has obvious absorption capacity of the UV light and VIS light and also has obvious absorption capacity in the NIR light range (λ > 780 nm). mTHPC/bCN and mTHPC/pCN not only maintain the absorption performance of bCN and pCN in the UV light and VIS light range, but also retain the absorption capacity of mTHPC in the NIR light range (λ > 780 nm) to some extent. Furthermore, the light absorption capacity of mTHPC/pCN is significantly better than that of mTHPC/bCN.

The calculated energy band gaps (*E*_g_) of mTHPC and pCN by the absorption spectra and the equation of α*h*ν = *A*(*h*ν – *E*_g_)^*n*/2^ (Li et al., 2018, 2019) are 1.45 and 2.45 eV in Figure 5B, respectively. The α, *h*, ν, *A, E*_g_, and *n* stand for the absorption coefficient, the Planck's constant, the light frequency, the proportionality constant, the energy band gap, and *n* = 1 for a direct band gap transition, respectively (Adhikari et al., 2017).

### Photocatalytic Hydrogen Evolution

**Figure 6:**
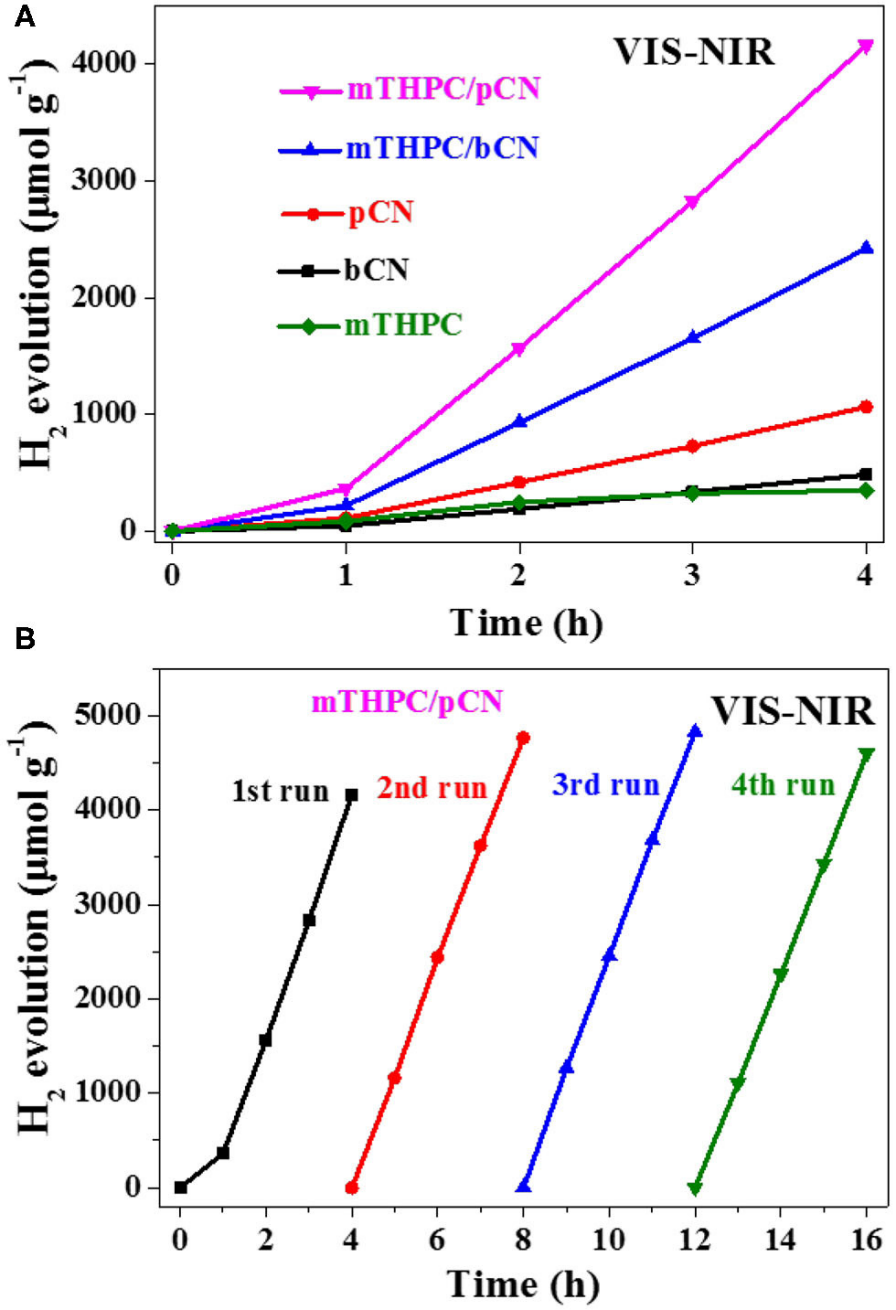
Photocatalytic hydrogen evolution activity from water of the five photocatalysts **(A)** and the cycle stability test of mTHPC/pCN **(B)** under VIS-NIR (λ > 420 nm) irradiation.

Photocatalytic hydrogen generation was performed under a 300-W xenon (Xe) lamp as the light source. Figure 6A shows the photocatalytic hydrogen production performance of samples under VIS-NIR light irradiation. A filter was used to get VIS-NIR light source (λ > 420 nm), 3 wt.% Pt was used as the co-catalyst, and TEOA was used as the sacrificial reagent. bCN and mTHPC show low hydrogen production performance; the average hydrogen evolution rates (HERs) are 120.6 and 87.4 μmol · h^−1^ · g^−1^, respectively.

The average HER of pCN and mTHPC/bCN are 266.0 and 605.8 μmol · h^−1^ · g^−1^, respectively. mTHPC/pCN shows the highest average HER of 1,041.4 μmol · h^−1^ · g^−1^, somewhat lower than the average HER obtained by Ce6/pCN (1,275.6 μmol · h^−1^ · g^−1^) (Liu et al., 2020a) and Pp/pGCN (1,153.8 μmol · h^−1^ · g^−1^) (Liu et al., 2020b). Furthermore, mTHPC/pCN has good cycle stability under VIS-NIR irradiation in Figure 6B. The XRD patterns of the fresh mTHPC/pCN and the used mTHPC/pCN are shown in Supplementary Figure 5. Compared with the fresh mTHPC/pCN, the peak intensities of the used mTHPC/pCN decrease to some extent, and the used mTHPC/pCN well maintains the crystal structure of the fresh mTHPC/pCN.

**Figure 7:**
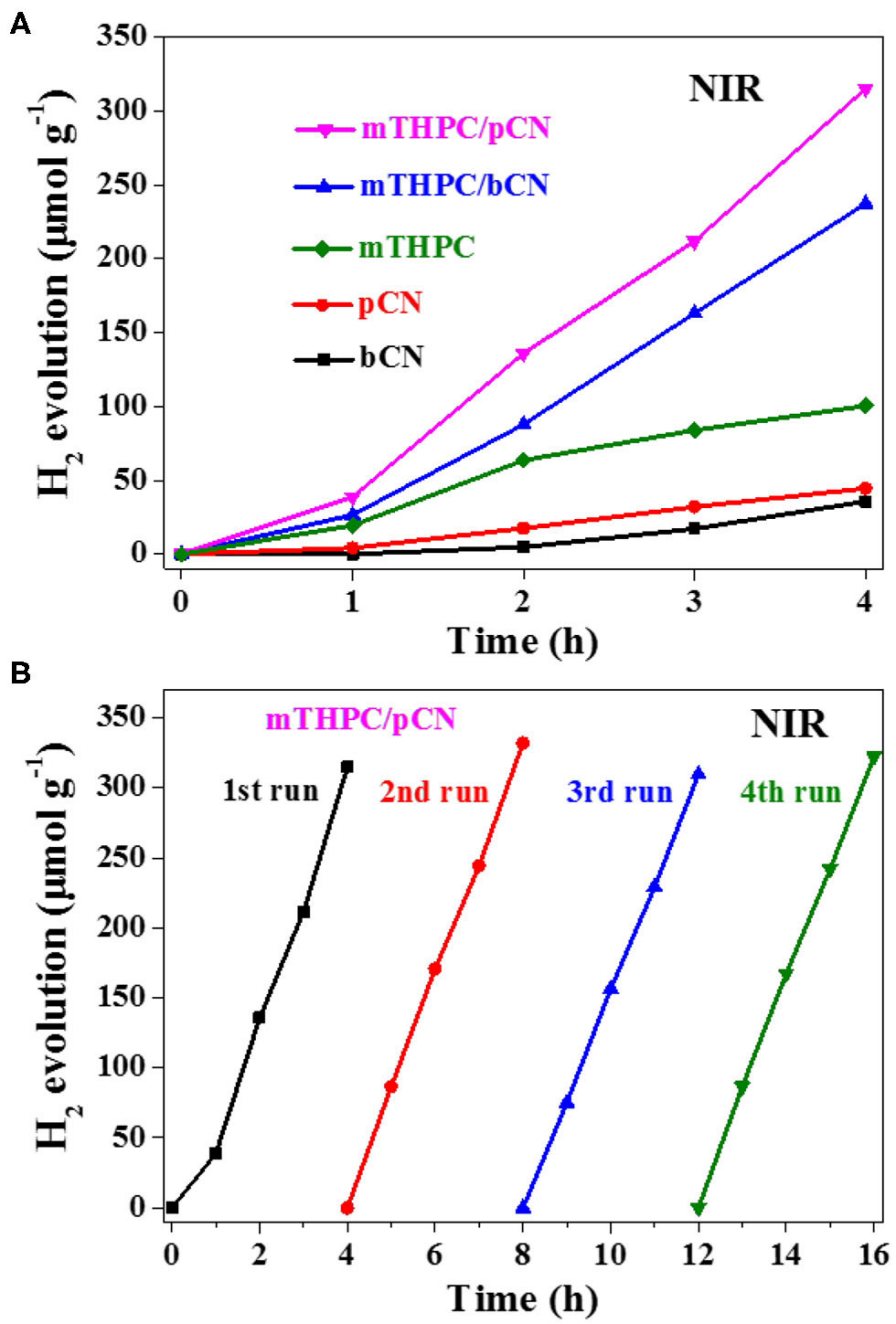
Photocatalytic hydrogen evolution activity from water of the five photocatalysts **(A)** and the cycle stability test of mTHPC/pCN **(B)** under NIR light (λ > 780 nm) irradiation.

Figure 7A shows the photocatalytic hydrogen production activity of samples under NIR light irradiation. A filter was used to get NIR light source (λ > 780 nm), Pt was used as the cocatalyst, and TEOA was used as the sacrificial reagent. bCN and pCN show trace amounts of hydrogen production, while mTHPC shows the low average HER of 25.1 μmol·h^−1^ · g^−1^.

The average HER of mTHPC/bCN is 59.3 μmol · h^−1^ · g^−1^. mTHPC/pCN shows the highest average HER of 78.8 μmol · h^−1^ · g^−1^, lower than the rate obtained by Ce6/pCN (312.6 μmol · h^−1^ · g^−1^) (Liu et al., 2020a) and Pp/pGCN (307.8 μmol · h^−1^ · g^−1^) (Liu et al., 2020b). This may be because the UV-VIS-NIR absorption capacity of mTHPC is lower than that of Ce6 and Pp (Supplementary Figure 6). In addition, mTHPC/pCN has good cycle stability under NIR irradiation in Figure 7B.

### EIS, Photocurrent Response Curves, ESR, and Phosphorescence Spectra

**Figure 8:**
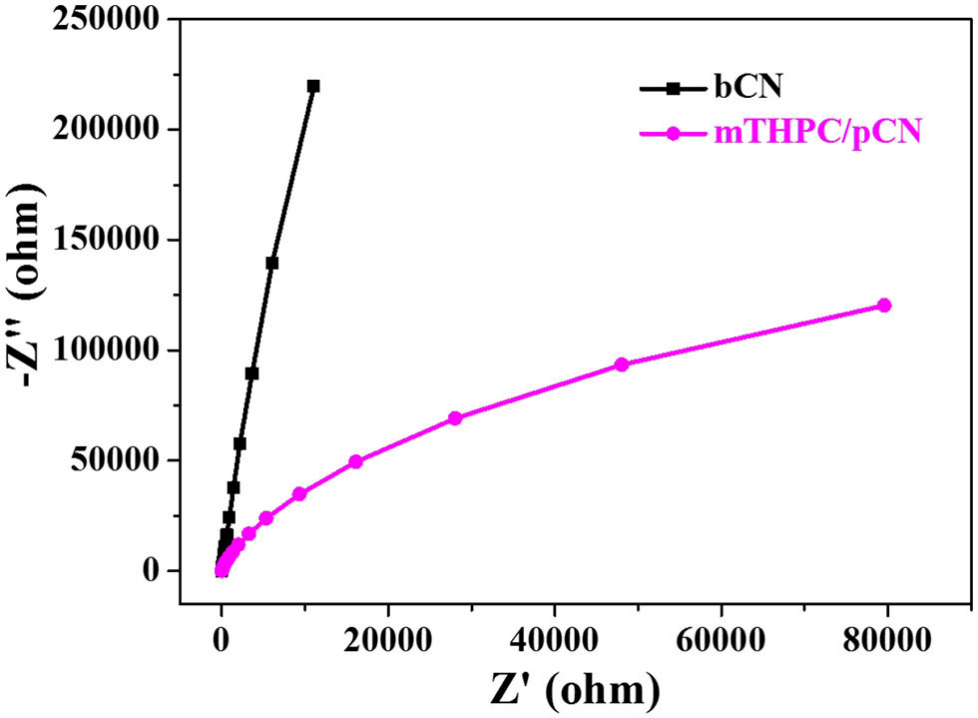
EIS of bCN and mTHPC/pCN.

Figure 8 shows the EIS comparison of bCN and mTHPC/pCN. The arc size in the high-frequency region of the Nyquist diagram is consistent with the electron transfer restriction mechanism, and the arc diameter is equal to the resistance of the electron transfer (She et al., 2016; Li S. J. et al., 2020). Obviously, the arc radius of mTHPC/pCN is significantly smaller than bCN, indicating that mTHPC/pCN can significantly retard the recombination of photogenerated electrons and holes and accelerate electron transfer (Li et al., 2017).

**Figure 9:**
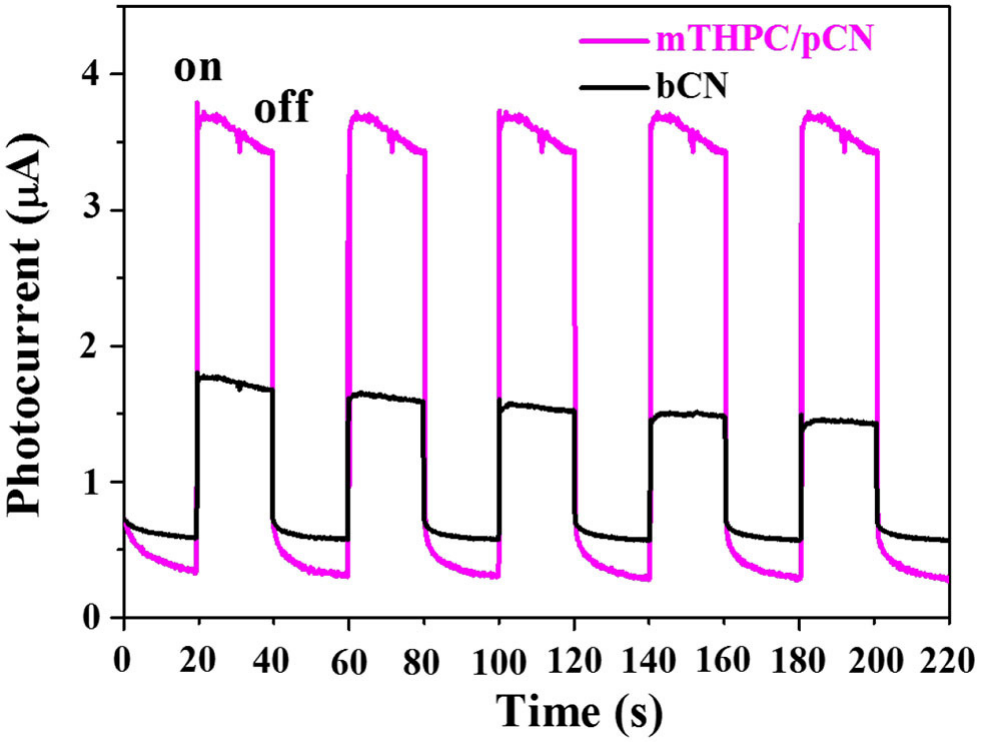
Photocurrent response curves of bCN and mTHPC/pCN.

Figure 9 shows the transient photocurrent response curves comparison of bCN and mTHPC/pCN under VIS-NIR (λ > 420 nm) irradiation. When turning on the light source, the photocurrents of the two samples rise immediately (Shen et al., 2020a). Conversely, when turning off the light source, the photocurrents drop quickly (Luo et al., 2018; Li et al., 2021). The pattern can be repeated, indicating that photogenerated electrons can be transferred to the contact interface through the sample under light irradiation (Tian et al., 2017; Shen et al., 2020b). Further observation found that the photocurrent value of mTHPC/pCN is higher than bCN, indicating that the charge separation efficiency of mTHPC/pCN has significantly enhanced (An et al., 2017).

**Figure 10:**
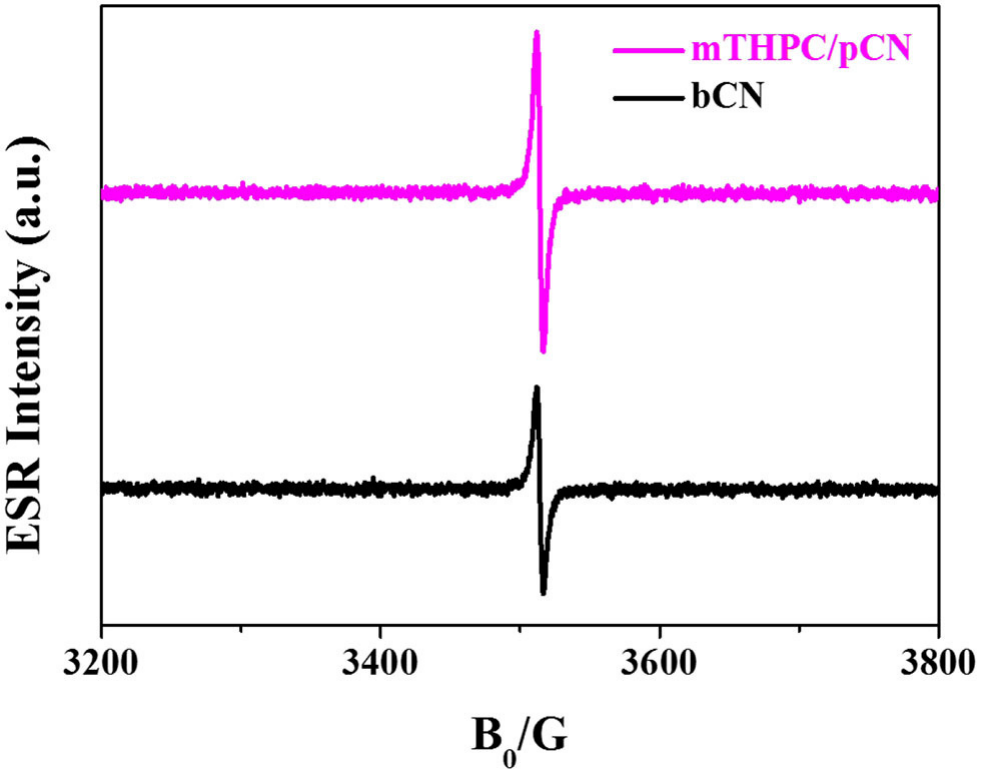
ESR of the bCN and mTHPC/pCN.

Figure 10 exhibits the ESR characterization of bCN and mTHPC/pCN under NIR light (λ > 780 nm) at room temperature. bCN and mTHPC/pCN both exhibit one single Lorentz line (*g* = 2.0034) from 3,200 to 3,800 G magnetic field (Liu G. et al., 2019; Jia et al., 2020). However, compared with bCN, the ESR intensity of mTHPC/pCN is much stronger, indicating that the concentration of unpaired electrons is much higher.

**Figure 11:**
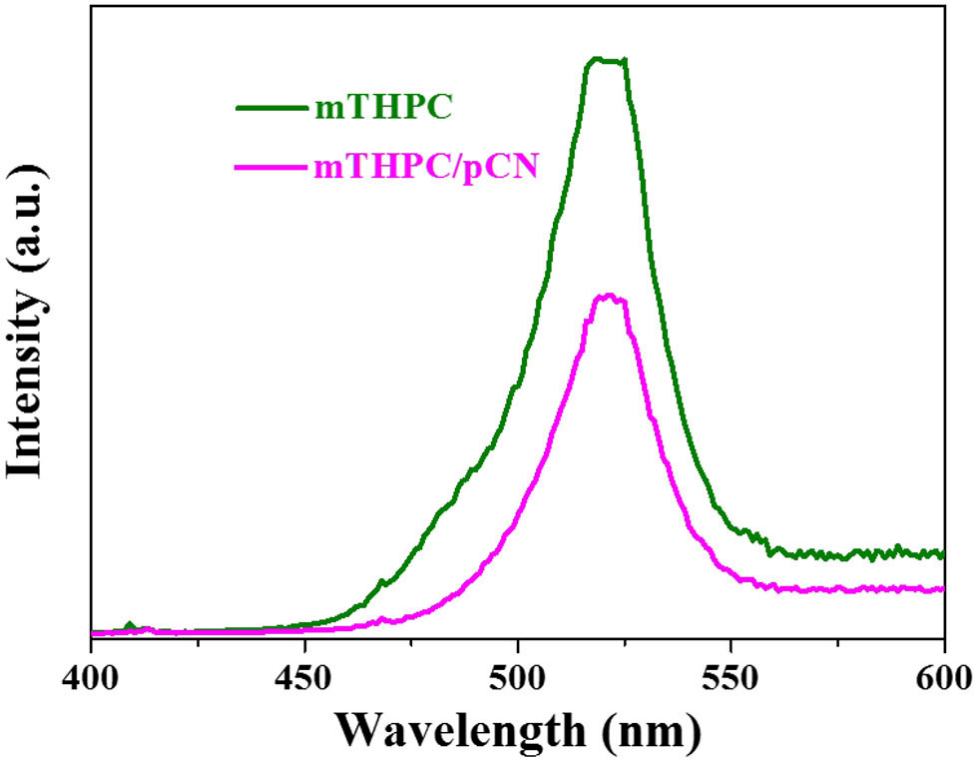
The phosphorescence spectra of mTHPC and mTHPC/pCN.

The phosphorescence spectra of mTHPC and mTHPC/pCN were tested at an excitation wavelength of 808 nm at room temperature in Figure 11. Obviously, the emission wavelength of mTHPC is in the range of VIS light (400–600 nm), indicating that the mTHPC has obvious up-conversion behavior (Wang et al., 2018). Compared with mTHPC, the emission peak intensity of mTHPC/pCN is significantly decreased, indicating the energy transfer from mTHPC to pCN.

### Photocatalytic Mechanism

The energy band gaps (*E*_g_) of mTHPC and pCN are 1.45 and 2.45 eV in Figure 5B, respectively. The XPS valence band (VB) top position of mTHPC and pCN are 0.62 and 1.29 V in Supplementary Figure 7, respectively. Thus, contrasting to the standard hydrogen electrode potential, the conduction bands (CBs) of mTHPC and pCN are −0.83 and −1.16 V, respectively.

**Figure 12:**
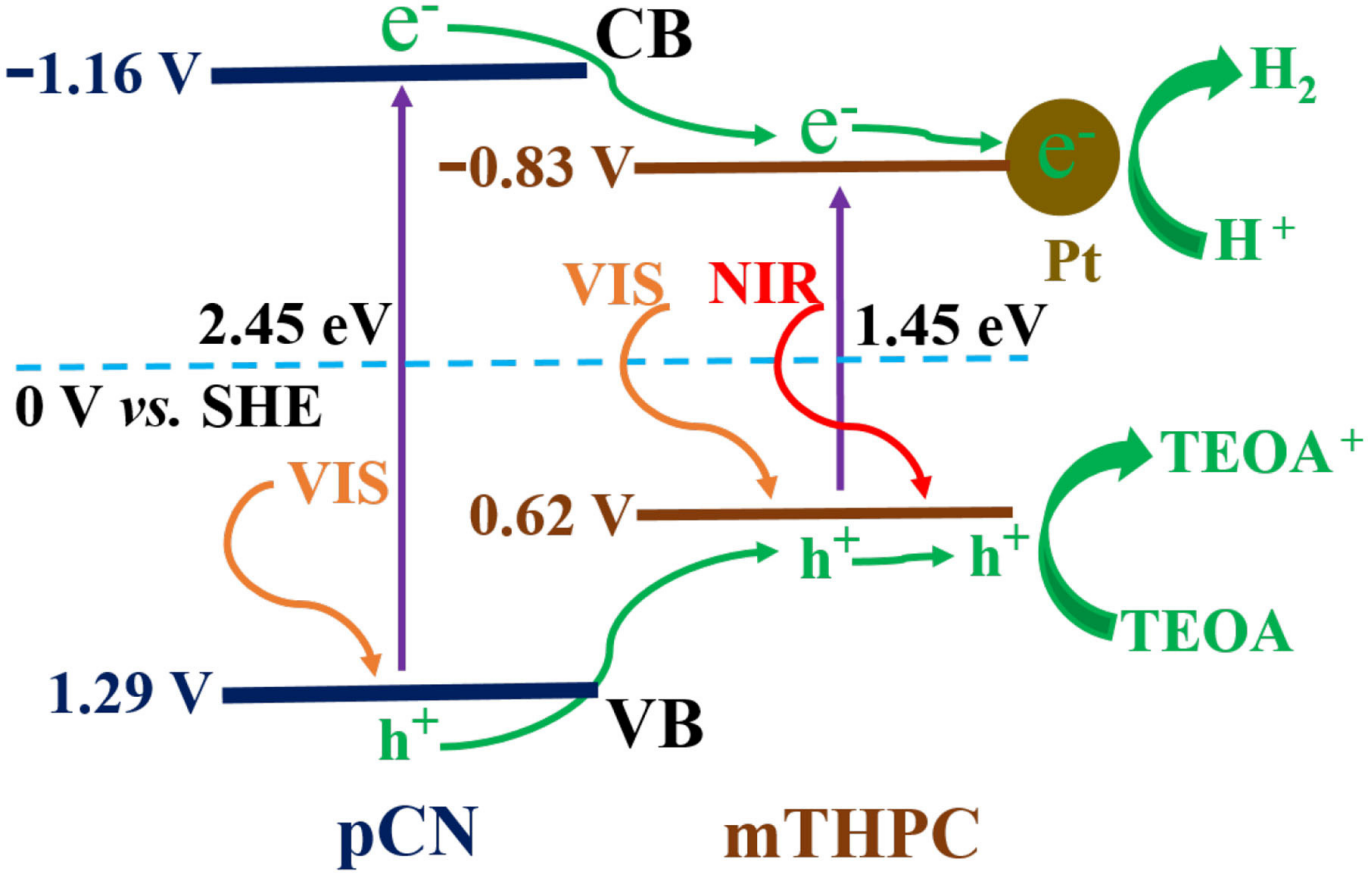
The photocatalytic mechanism of mTHPC/pCN.

**Figure 13:**
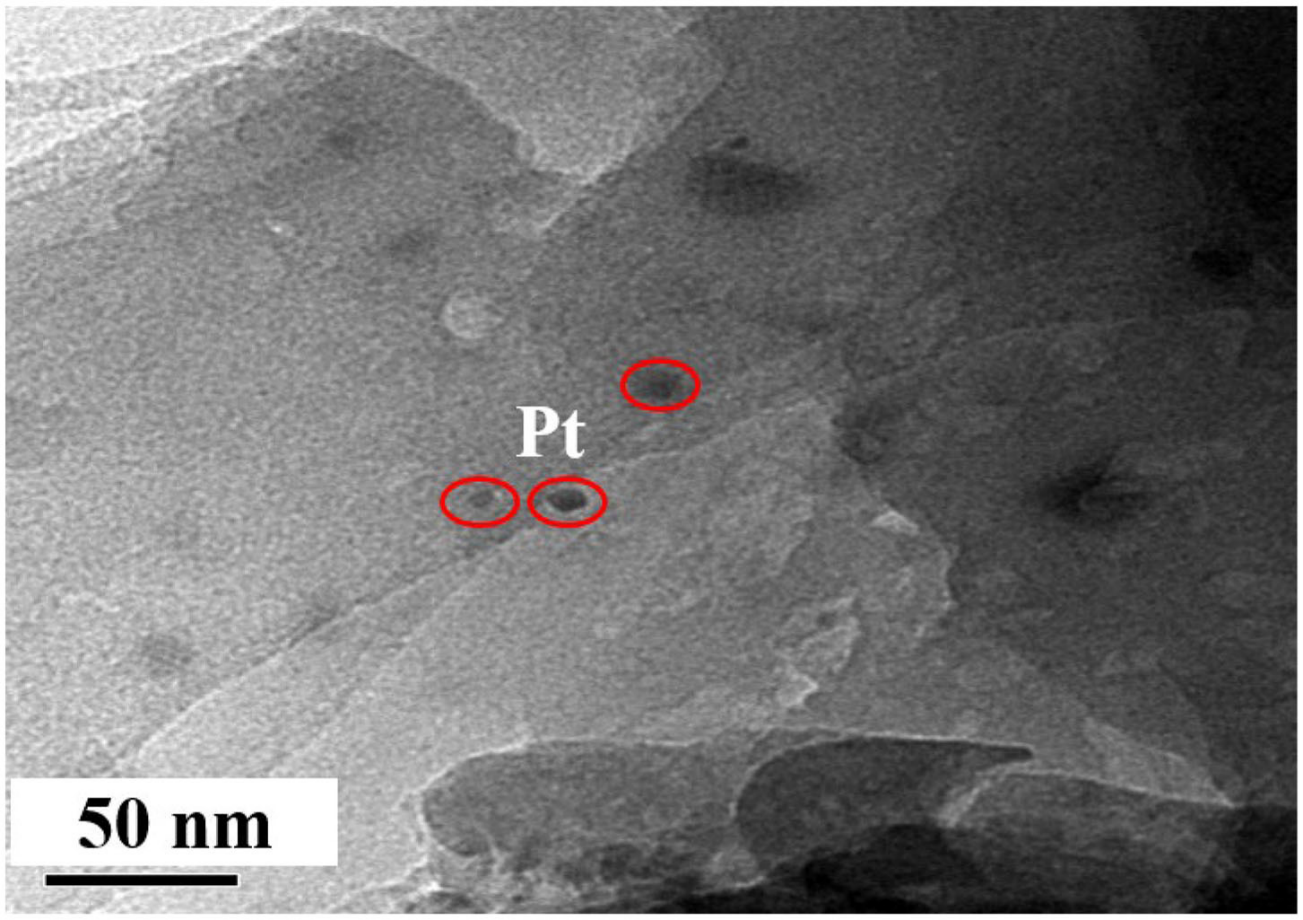
The TEM image of mTHPC/pCN collected after conducting photocatalytic reaction for one time.

A possible mechanism for mTHPC/pCN is proposed in Figure 12. When mTHPC/pCN is irradiated under VIS light, pCN is excited to generate electrons (e^−^) on CB and holes (h^+^) on VB. Because the CB edge potential of pCN (−1.16 V) is more negative than that of mTHPC (−0.83 V), the e^−^ on pCN could transfer to the CB of mTHPC. Pt was used as the cocatalyst (Figure 13 and Supplementary Figure 8) obtained from the Pt precursor (H_2_PtCl_6_ · 6H_2_O) by the *in situ* photoreduction (Sui et al., 2013; Pan et al., 2017). Pt nanoparticles may slightly enhance absorption intensity in NIR region (Supplementary Figure 9) due to the light scattering phenomenon of Pt (Shiraishi et al., 2014; Chen et al., 2020). Pt can quickly transfer e^−^ which can reduce H^+^ to H_2_ (Xing et al., 2014; Zhou et al., 2019). Indeed, the photocatalytic hydrogen evolution performance is significantly enhanced (Supplementary Figure 10). In addition, because the VB of pCN (1.29 V) is more positive than that of mTHPC (0.62 V), the h^+^ on pCN could transfer to the VB of mTHPC. As the sacrificial agent, TEOA can quickly transfer h^+^ and be used as TEOA to TEOA^+^ (Xing et al., 2014; Zhou et al., 2019).

When mTHPC/pCN is irradiated under NIR light, mTHPC has an up-conversion behavior (Figure 11); i.e., the irradiation of mTHPC under NIR light can generate VIS light for pCN to work. Actually, the ESR characterization showed the concentration of unpaired electrons is much higher in mTHPC/pCN than bCN under NIR light irradiation (Figure 10). In addition, although under NIR light irradiation only, mTHPC/pCN still works because of its NIR absorption capacity (Figure 5A) and the up-conversion behavior of mTHPC (Figure 11).

## Conclusions

mTHPC/pCN prepared by positively charged pCN was modified by negatively charged mTHPC for the first time. mTHPC/pCN can allow for efficient charge separation and transfer and retard the recombination of photogenerated electrons and holes. In addition, mTHPC/pCN has a wide range of VIS light and NIR light absorption capabilities and thus the enhanced photocatalytic hydrogen evolution performance and good stability. The current results show that using a photosensitizer can enhance the light absorption intensity of the VIS light–driven g-C_3_N_4_ system and expand the absorption and utilization of the solar spectrum range. This work provides some new insights and directions for the realization of photocatalytic hydrogen evolution under VIS/NIR light region.

## Data Availability Statement

The raw data supporting the conclusions of this article will be made available by the authors, without undue reservation.

## Author Contributions

YL came up with the idea, designed the experiments, analyzed the data, and wrote the paper. ZM guided the research and revised the paper. Both authors contributed to the article and approved the submitted version.

## Conflict of Interest

The authors declare that the research was conducted in the absence of any commercial or financial relationships that could be construed as a potential conflict of interest.
