# Supplementary material for: g-C3N4 Modified by meso-Tetrahydroxyphenylchlorin for Photocatalytic Hydrogen Evolution Under Visible/Near-Infrared Light
Source: Front Chem. 2020 Nov 6;8:605343. doi: 10.3389/fchem.2020.605343 (PMC7677346; doi:10.3389/fchem.2020.605343)
Supplement: Supplementary file 1 [file Table_1.DOCX]

Supplementary Material

Table S1. Specific surface areas and the pore volumes of samples

| Samples | SSA (m^2^·g^−1^) | Pore volume (cm^3^·g^−1^) |
| --- | --- | --- |
| bCN | 12.5 | 0.063 |
| pCN | 40.3 | 0.163 |
| mTHPC/bCN | 10.9 | 0.056 |
| mTHPC/pCN | 30.8 | 0.126 |


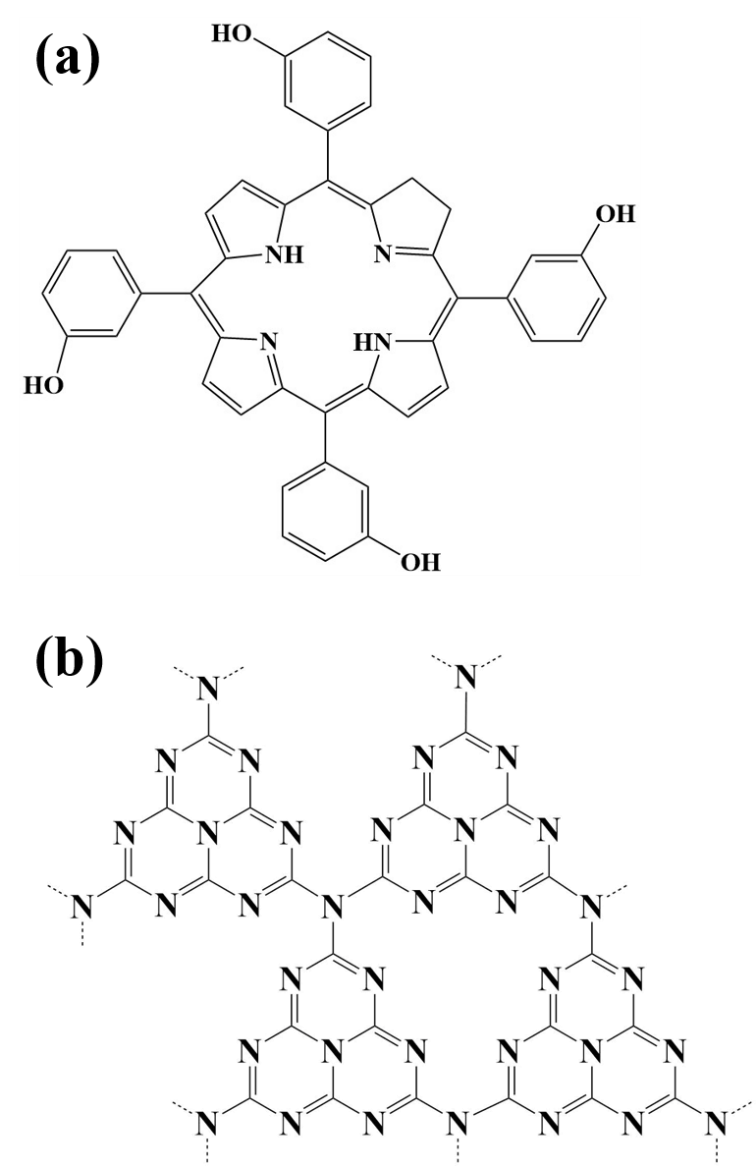


Fig. S1. Chemical structures of mTHPC (a) and bCN (b).


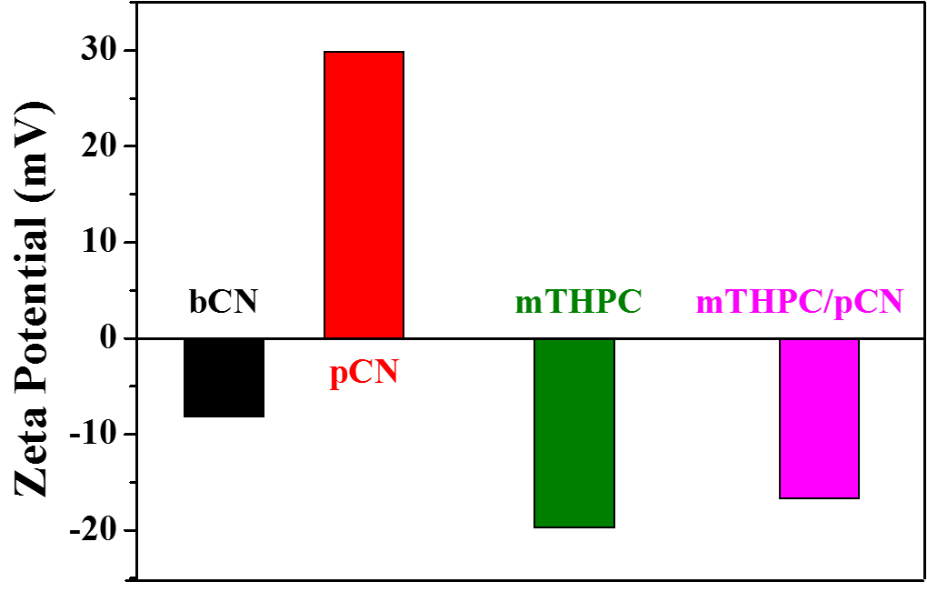


Fig. S2. Zeta potentials of samples dispersed in deionized water.


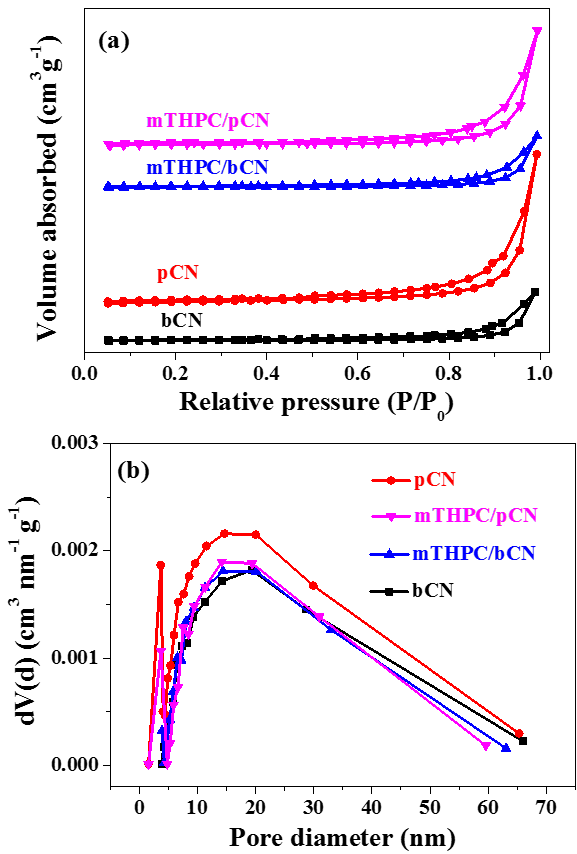


Fig. S3. N_2_ adsorption-desorption isotherms (a) and the corresponding pore size distribution curves (b) of samples.


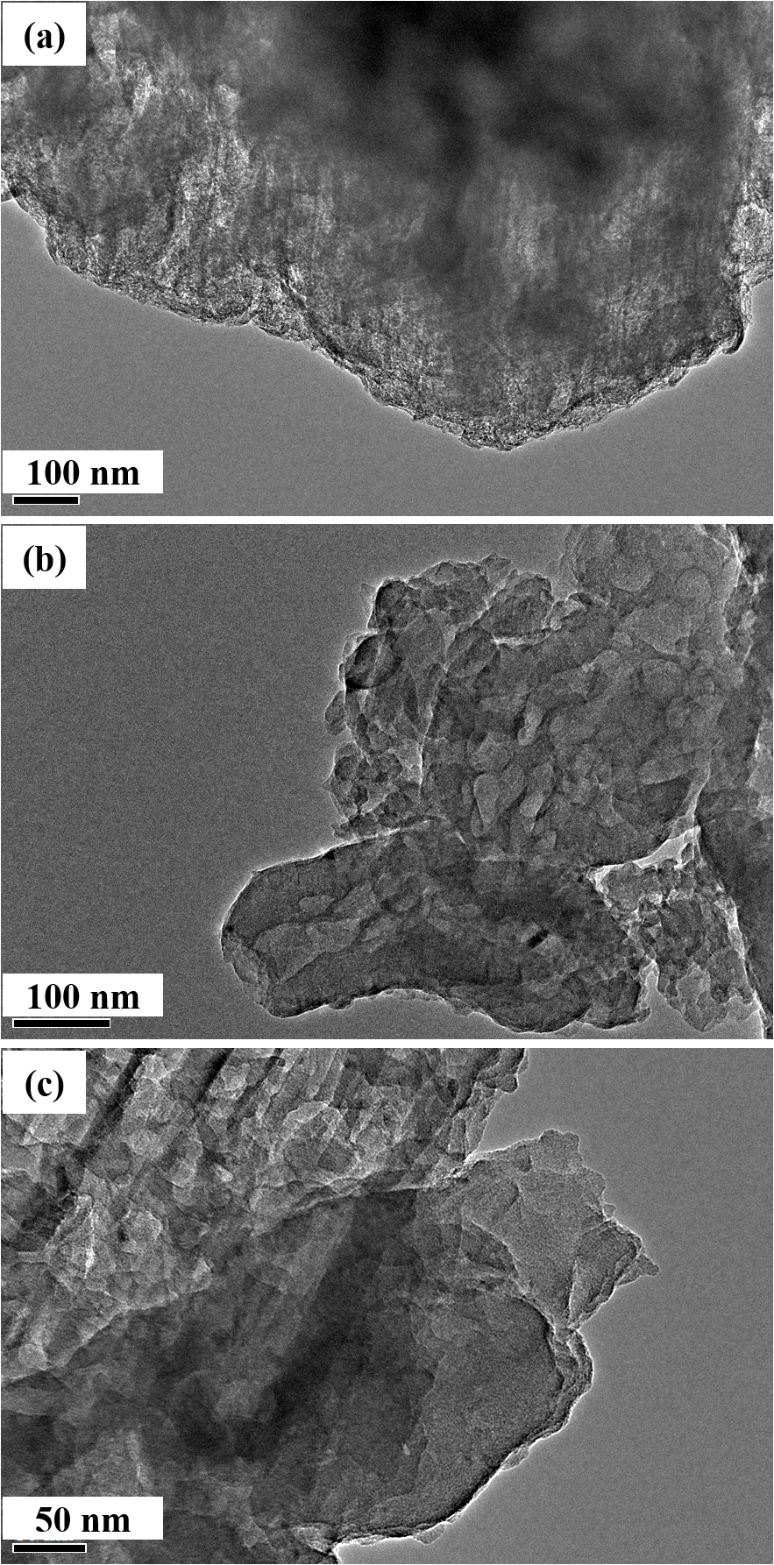


Fig. S4. TEM images of bCN (a), pCN (b), and mTHPC/pCN (c).


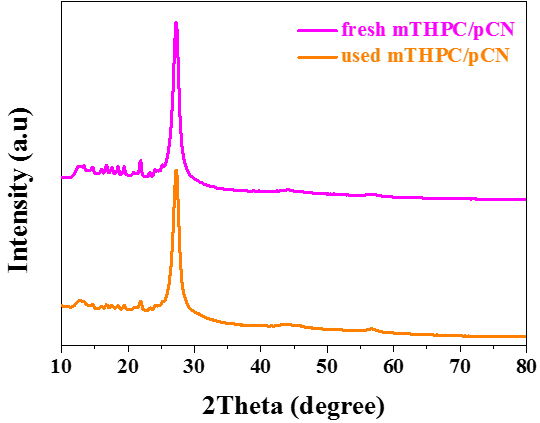


Fig. S5. XRD patterns of the fresh mTHPC/pCN and the used mTHPC/pCN.


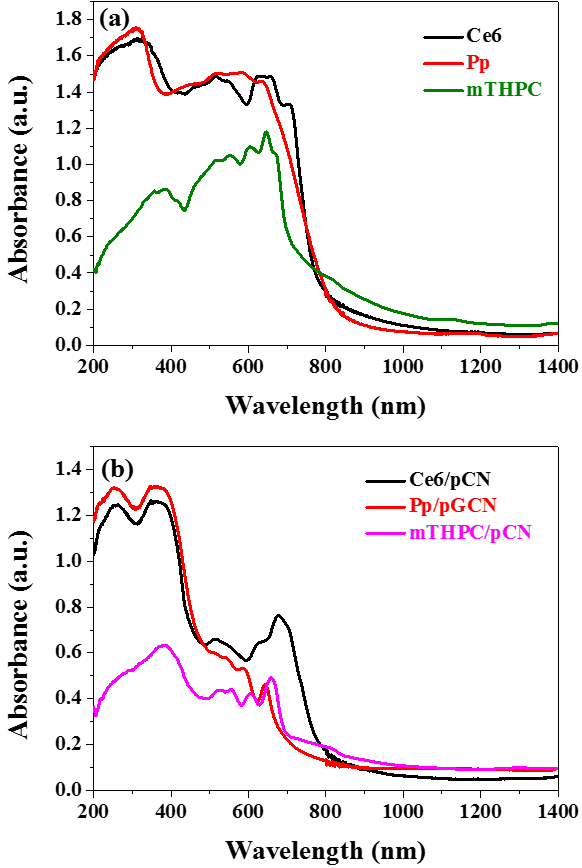


Fig. S6. UV-Vis-NIR spectra of Ce6, Pp, mTHPC (a) and Ce6/pCN, Pp/pGCN, mTHPC/pCN (b).

**
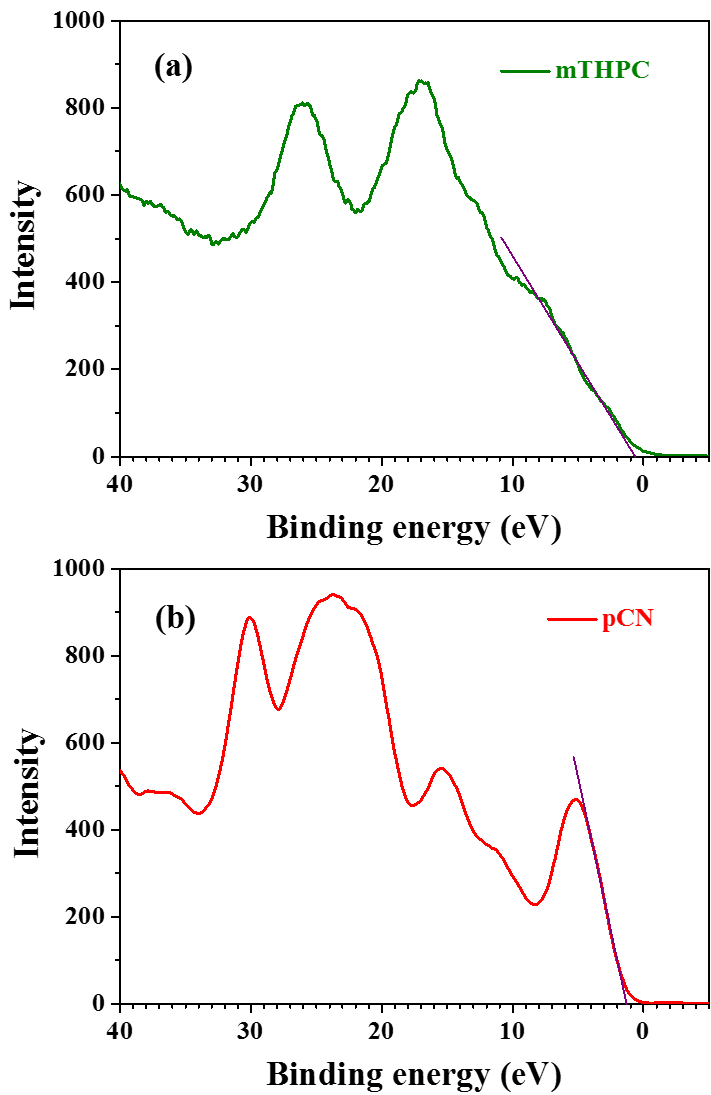
**

Fig. S7. XPS valence band (VB) spectra of mTHPC (a) and pCN (b).


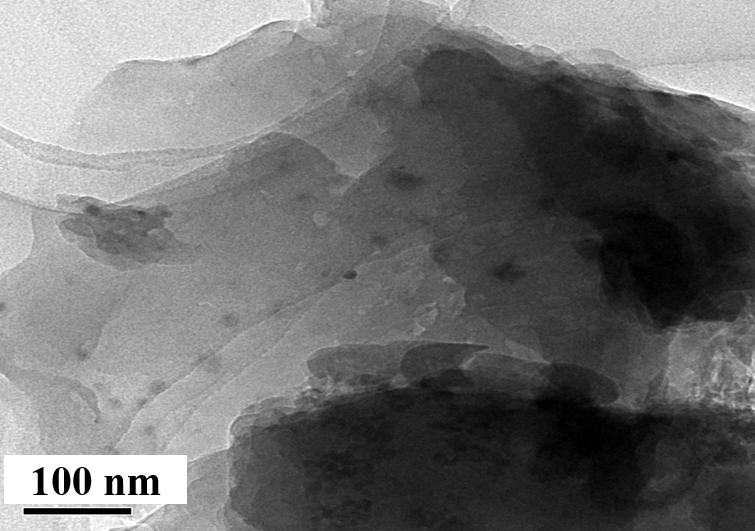


Fig. S8. TEM image of mTHPC/pCN collected after one photocatalytic reaction.


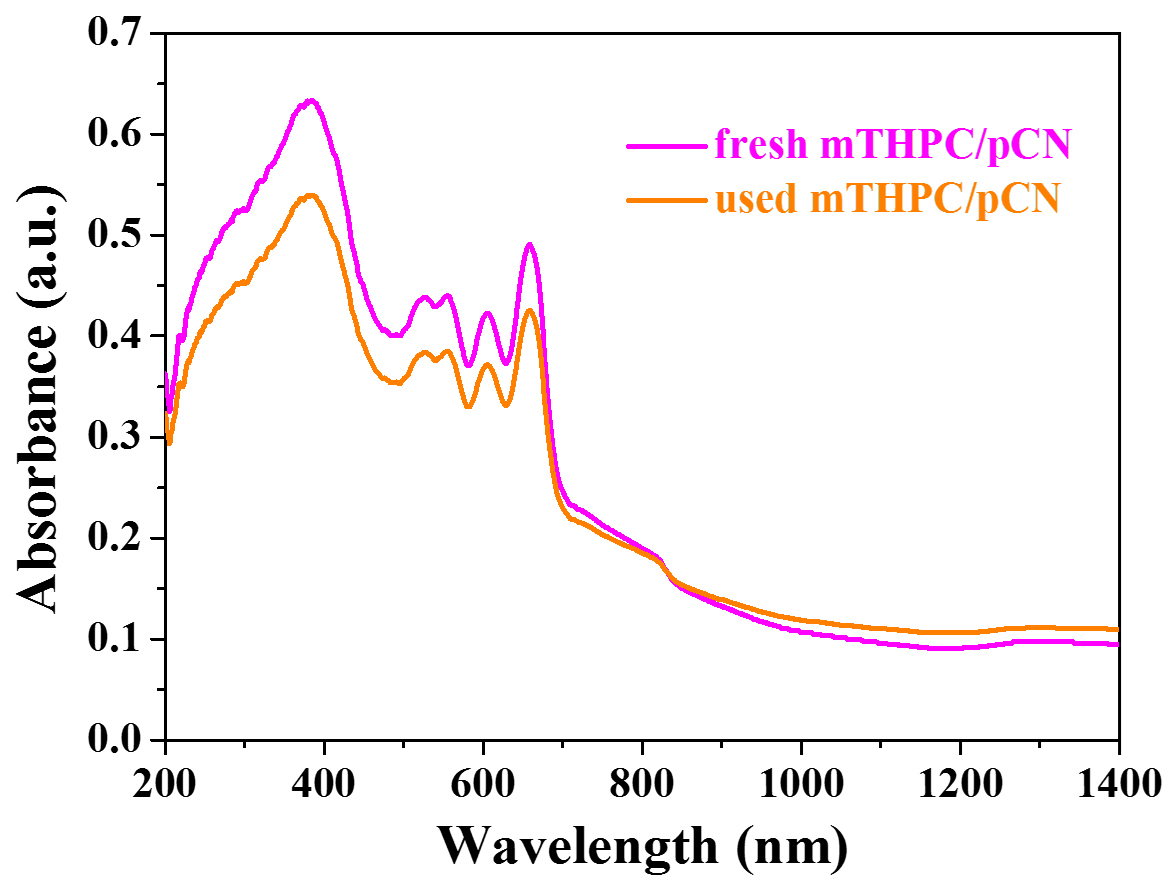


Fig. S9. UV-VIS-NIR absorbance spectra of the fresh mTHPC/pCN and the used mTHPC/pCN collected after once photocatalytic reaction.


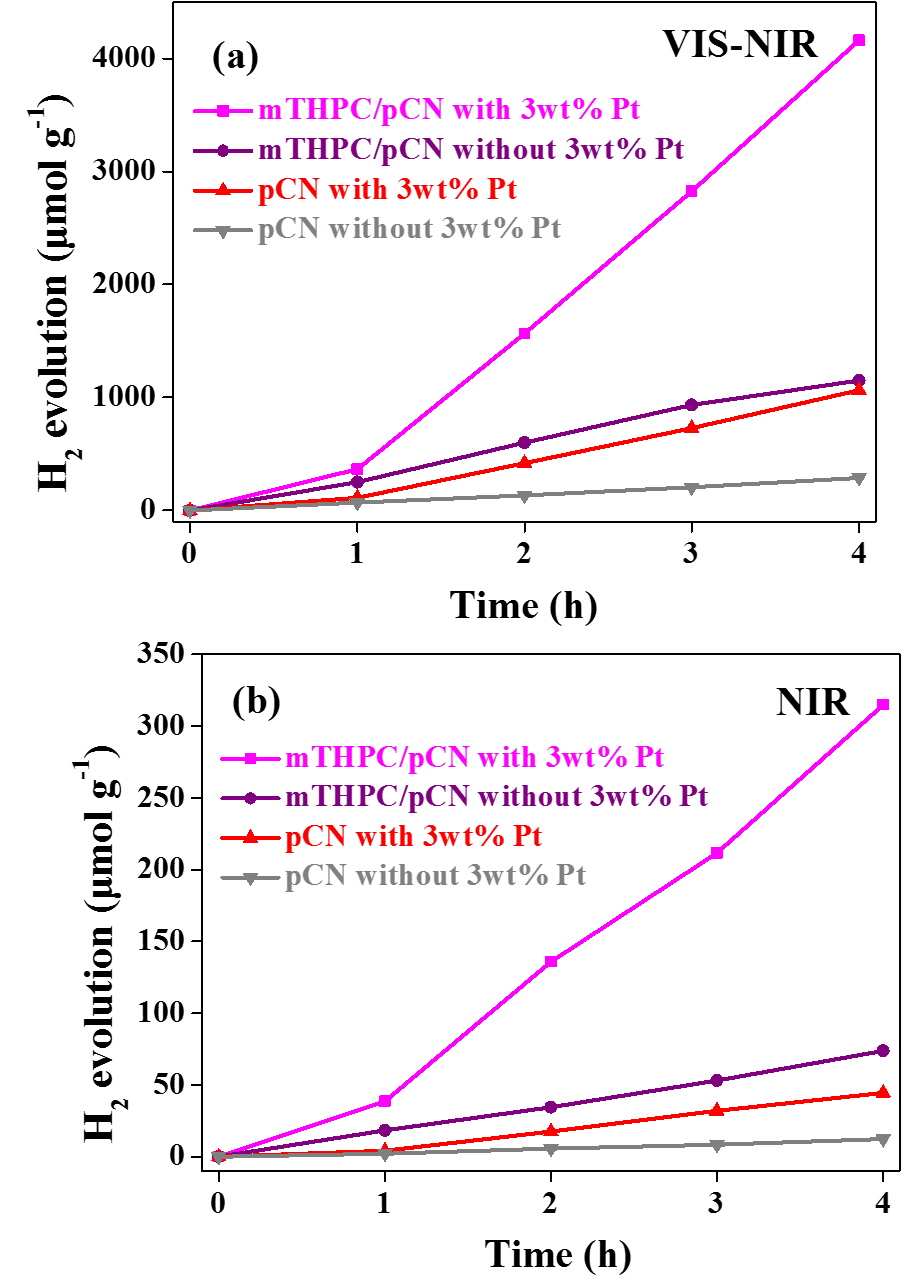


Fig. S10. Photocatalytic hydrogen evolution activity under VIS-NIR light (λ > 420 nm) irradiation (a) or under NIR light (λ > 780 nm) irradiation (b).
